# Loss of Obstetric Services in Rural Appalachia: A Qualitative Study of Community Perceptions

**DOI:** 10.13023/jah.0302.02

**Published:** 2021-05-03

**Authors:** Caroline R. Efird, David Dry, Rachel F. Seidman

**Affiliations:** Department of Health Behavior, Gillings School of Global Public Health, University of North Carolina at Chapel Hill; Department of History, University of North Carolina at Chapel Hill; Center for the Study of the American South, University of North Carolina at Chapel Hill

**Keywords:** Appalachia, rural health, access to care, obstetrics, qualitative research

## Abstract

**Background:**

As rural hospitals across the United States increasingly downsize or close, the availability of inpatient obstetric services continues to decline in rural areas. In rural Appalachia, the termination of obstetric services threatens to exacerbate the existing risk of adverse birth outcomes for women and infants, yet less is known about how the cessation of these services affects the broader community.

**Purpose:**

The purpose of this paper is to explain how the loss of local obstetric services affects perceptions of healthcare among multi-generational residents of a remote, rural Appalachian community in western North Carolina.

**Methods:**

An interdisciplinary team of researchers conducted a thematic analysis of health-related oral history interviews (n=14) that were collected from local residents of a rural, western North Carolina community during the summer of 2019.

**Results:**

The closure of a local hospital’s labor and delivery department fostered (1) frustration with the decline in hospital services, (2) perceived increases in barriers to accessing healthcare, and (3) increased medical mistrust.

**Implications:**

Findings suggest that the loss of obstetric services in this rural Appalachian community could have broad, negative health implications for all residents, regardless of their age, sex, or ability to bear children. Community-specific strategies are needed to foster trust in the remaining healthcare providers and to increase access to care for local residents. Results serve as formative research to support the development of interventions and policies that effectively respond to all community members’ needs and concerns following the loss of obstetric services in remote Appalachian communities.

## BACKGROUND

A wave of rural hospital closures and the elimination of obstetric departments in many surviving rural hospitals has decreased the availability of hospital-based obstetric care for rural residents.^1^,^2^ Between 2004 and 2014, over 179 rural counties in the U.S. lost hospital-based obstetric services, a decline of 9%.^3^ This loss of services poses potential health risks to rural women and infants, as the need to drive long distances for obstetric services has been associated with adverse maternal and infant birth outcomes.^4^,^5^ Elimination of hospital-based obstetric services in rural areas can also increase out-of-hospital births and births in hospitals without adequate neonatal equipment.^6^–^8^ In addition, cessation of rural obstetric care is linked to increases in unplanned inductions and high-risk cesarean deliveries.^2^,^6^–^8^

In rural Appalachian counties, the termination of obstetric services threatens to exacerbate existing health disparities, as women across the region experience elevated risk for pregnancy-related morbidity and mortality.^9^ According to a recent health report from Appalachian Regional Commission (ARC), 25% of the counties that comprise the Appalachian Region are considered rural.^10^ The ARC notes that infant mortality in Appalachia is 16% higher than the rest of the nation.^10^ In particular, the southern Appalachian subregion has an infant mortality rate of 7.4 per 1000 births, or 21% higher than the national average.^10^ In a region where women and infants already experience increased risk of adverse birth outcomes, the loss of obstetric services has the potential to perpetuate regional health disparities.

Researchers have identified the need for qualitative studies to “provide nuanced insight into sociocultural and health service factors that impact rural obstetrical care” in Appalachia.^5^ While it is critical to document the effects of loss of obstetric services on women of childbearing age in rural Appalachian areas,^5^ less is known about how the termination of these services affects all community members’ healthcare-related attitudes and beliefs. Documenting the sociocultural and structural factors that uniquely influence rural Appalachian residents’ perceptions of care is foundational to ensuring that people in these areas receive the healthcare services that they need.^11^ Thus, this paper seeks to document how the loss of local obstetric services affects perceptions of health care among residents of a rural Appalachian community in western North Carolina (NC).

## METHODS

### Study Design and Setting

This analysis is based on oral history interviews (*n*=14) that were collected in western NC during summer 2019, as a subproject of a larger oral history collection study that aimed to capture the diverse health and healthcare experiences of North Carolinians who live in rural areas. These interviews were conducted 2 years following the closure of a labor and delivery department at a hospital that primarily serves two mountainous, Appalachian counties with a combined population of approximately 33,000.^12^ The Office of Human Research Ethics at the sponsoring university reviewed this project and determined that it did not constitute human subjects research as defined under federal regulations.

Interview questions encompassed broad topics related to life history, community health, and interactions between healthcare providers and patients. Oral history diverges from traditional in-depth qualitative interviewing^13^ in that interviewees have shared authority with the researchers in several areas: (1) interview topics, (2) accuracy of the transcript, and (3) archival of the interview.^14^ For example, interviewees were invited to discuss topics that were of interest to them in addition to the questions on the interview guide. Audio was professionally transcribed and mailed to interviewees for their approval. Further, all participants provided written consent that interview audio and transcripts could be stored within a public archive. To protect the identity of participants who were employed at the local hospital, all identifying information is excluded in these findings.

### Data Collection

Project staff collaborated with community partners in western NC to purposely recruit long-term and lifetime residents (*n*=14) of rural Appalachia. Because the larger research study aimed to capture patient and provider experiences of health and health care, seven interviewees were practicing or retired healthcare providers (including nurses, physicians, a midwife, and a clinical social worker). Previous health-related qualitative studies in rural communities^11^,^15^–^17^ have shown that a sample size of 14 is sufficient to reach theoretical saturation within the data.^11^,^18^ All interviewees self-identified as white and non-Hispanic and ranged in age from 29 to 90 years. There were an equal number of male and female interviewees.

### Data Analysis

Closely following Braun and Clarke’s^19^ approach to thematic analysis, the authors became familiar with the data by listening to 19 hours of interview audio and reading all transcripts. Analytic memos^20^ were produced to document the health-related content in each interview. A codebook consisting of inductive and deductive codes^20^ was developed and ATLASti qualitative analytic software was used. After meeting regularly to review and discuss the coding processes and results, data matrices were created (Table 1) to summarize the findings from salient codes and to develop related themes that were present across interviews.

## RESULTS

The thematic analysis produced three primary themes: across healthcare providers and nonproviders, interviewees’ responses to the termination of obstetric services at the rural hospital included (1) frustration with decline in hospital services, (2) perceived increases in barriers to accessing health care, and (3) medical mistrust. Quotes from interviewees who were practicing or retired healthcare providers are preceded with the designation of “provider.”

### Frustration with Decline in Hospital Services

To access obstetric services, residents indicated that community members must now travel 30 to 50 miles through mountainous terrain to reach an urban hospital. Only one interviewee was a woman of childbearing age, yet providers and nonproviders repeatedly described the loss of the local labor and delivery department as a critical threat to the health in their community:

Provider:To think that the dangers for people having a healthy and safe delivery [in our community] have gone backward—I can’t put into words how shocked I am that medical care was better 45 years ago [when I moved here].

Providers also expressed anxiety that patients did not understand the diminished capacities of the local hospital and the need to travel to an urban hospital for prenatal care and delivery. Pregnant women still frequented the local hospital and had to be turned away or transferred. Despite the lack of obstetric services, laboring women had recently given birth in the emergency department of the local hospital.

Provider: The challenge is getting people to understand, especially pregnant young women, that, yes, you can come [to local hospital] but you *need* to go to a facility that has the ability to monitor this baby…[local hospital] is not the best choice anymore, but it used to be.

Additionally, interviewees linked the closing of the labor and delivery department to the decline of other services. Providers argued the termination of obstetrics made the cost of offering 24-hour anesthesia coverage impractical for the local hospital, and the loss of anesthesia led to the outflow of the orthopedics and surgery departments. Therefore, interviewees suggested the loss of obstetrics subsequently increased the health vulnerabilities of the entire community, not just that of women and infants.

[My spouse and I] are through having babies, but our friends’ children aren’t through, or our neighbors...our whole larger community in the mountains has a deep threat. It has a deep threat to health. And it’s not just [obstetrics] and mother and childcare, it’s across the board.

Thus, providers and nonproviders indicated that the loss of obstetric services was associated with the overall decline in services at the local hospital, which contributed to their sense of increased health risks for all community members.

### Perceived Increases in Barriers to Accessing Health Care

Providers and nonproviders associated the termination of obstetrics with increasing transportation and cost barriers to accessing health care. They noted increased risks for pregnant community members as a result of the greater time and distance required to drive longer distances through potentially hazardous mountain terrain:

Provider: We can’t go to [local hospital] to deliver a baby…so it’s an hour to get to the [urban] hospital. A lot of things can happen in an hour. And in all cases of leaving this county, you drive through large stretches where there are no people. There’s no houses. There’s just woods, woods, woods, and curvy roads. And let’s just say it’s wintertime [when weather creates dangerous driving conditions].

In the face of endemic poverty in their region of Appalachia, providers and nonproviders also stressed how traveling long distances also resulted in increased transportation costs and lost wages, with longer travel times necessitating more time off work. Nonproviders expressed frustration at the cost of having to make multiple trips for follow-up appointments or referrals to various specialists. Interviewees described difficulties balancing their work schedules with the limited availability of urban providers and complained about long wait times to see urban providers.

Provider: It’s a financial burden that they can’t get [to urban hospital], which I have heard so many times [at the rural hospital]. ‘I don’t have the gas money to get to there.’

Overall, interviewees suggested that longer travel times to urban hospitals compounded pre-existing community transportation issues and increased the cost of obtaining obstetrical and other hospital-related medical care. These losses appeared to fuel a growing mistrust of providers and the broader healthcare system.

### Medical Mistrust

Interviewees who were not healthcare providers distinguished between local, rural providers and providers who lived and worked in urban areas. They expressed trust in local providers as known and accountable to patients as fellow community members. Many also regarded urban physicians as more monetarily motivated than rural physicians.

Doctors who get their medical degree and move to the country are there to help people. They’re not there to make money, or they’d have stayed in the city.

With the need to visit distant providers and resulting absence of community accountability and personal relationships with providers, interviewees who were not healthcare providers perceived that most urban providers prioritized financial profit over caring for patients.

Interviewees also argued that the lack of locally-available obstetric care limited opportunities for pregnant women to develop trust with providers. Local women increasingly gave birth with providers with whom they had not formed personal relationships or even met before going into labor, which some argued could make giving birth a more traumatic experience:

Provider: Making love and being raped are different, but they’re the same item in the same part of your body, but because of your emotional connection, it’s very different. So why is it different when someone’s in labor and someone shows up and pokes around who she’s never seen before and she has no relationship with, compared to somebody she’s seen during her pregnancy and developed trust with?

This sentiment mirrored the experiences of other interviewees, who reiterated the importance that members of their community placed on knowing and trusting their providers.

Further, interviewees perceived that the decision to close the obstetrics department was detrimental to community interests, and the decision generated widespread mistrust in the local hospital and the healthcare system in general. Providers and nonproviders suggested that the department’s closing was undertaken to make the nonprofit hospital system more attractive for its subsequent purchase by a national for-profit entity. As a result of its transfer in ownership, interviewees increasingly viewed the local hospital as less accountable to their community.

Provider: One of the wonderful things here, and this is maybe true of living in any small community, is that the medical care is personal. Now, we’ve just lost our hospital…I knew so many of the nurses, I knew their families. It was just so sweet...that’s changing, and I hate to see that change.

Because the local hospital was purchased by a for-profit company, interviewees spoke of “losing our hospital” as if the entire hospital had closed.

Interviewees highlighted wide-spread community dissatisfaction with the cessation of obstetric services, with one nonprovider stating “95% of people have not been happy” with the decision. They upheld the elimination of the labor and delivery department as evidence the hospital was no longer “independent” and free to put the interests of the local community at the forefront. They described local health care prior to the termination of obstetrics as more comprehensive, personalized, and affordable. Thus, they explicitly connected the closure of the labor and delivery department to a for-profit entity’s purchase of the local hospital, which influenced their mistrust of healthcare providers and systems.

## DISCUSSION

These findings indicate that regardless of interviewees’ individual or familial need for maternal or infant healthcare services, residents of this rural Appalachian community perceived that maternal and infant health were negatively affected by the cessation of obstetric services at their local hospital. Their perceptions are consistent with prior research, which indicates that loss of local access to obstetric services exacerbates the risk of adverse birth outcomes in rural communities.^3^,^5^ Interviewees believed that the termination of obstetrics worsened region-specific geographic and socioeconomic barriers related to accessing pregnancy-related care^21^ and other critical care services which were downsized following the closure of the labor and delivery department. Yet, interviewees’ discontent with the loss of obstetric services went beyond frustration with the financial burden of increased travel distance or trepidation about negative birth outcomes.

The findings presented in this article illuminate that interviewees associated the discontinuance of obstetric services with a for-profit company’s purchase of the local hospital, which influenced their general skepticism of the hospital and mistrust of healthcare providers who were not from the region. The purchase of the local hospital by a nonlocal, for-profit entity is an example of what sociologist David Walls describes as “internal colonialism,” a phenomenon that has occurred in Appalachia for decades when dominant, outside industrial entities establish control in the region and prevent autonomous development and progress.^22^ Viewed through an internal colonialism lens, 22 an event such as the hospital’s purchase, and the subsequent termination of the labor and delivery department, could foster rural Appalachian residents’ distrust of people who are perceived as outsiders.

Local media outlets documented residents' initial frustration concerning the department's closure, and the analysis in this manuscript provides an in-depth look into how, over time, residents’ initial dissatisfaction evolved into heightened medical mistrust in the community. Older adults in rural areas commonly mistrust healthcare providers,^23^ yet the loss of obstetric services appeared to intensify the nonproviders’ suspicions of providers who they did not consider to be members of their local community. Mistrust of providers is associated with hesitancy to seek medical care and underutilization of health services.^24^,^25^ Since neglecting to seek medical care puts rural residents at increased risk for medical complications and poor overall health,^23^,^26^ it may be especially harmful for residents of remote Appalachian communities who are already at risk for many adverse health outcomes.^27^ Therefore, within this rural Appalachian community, the loss of local obstetric services could have broad, negative health implications for many members of the community, regardless of their age, sex, or ability to bear children. These findings could help urban providers understand the importance of building trust with patients who come to them from rural communities in Appalachia.

To adequately address community members’ needs in rural Appalachian areas that have recently experienced the cessation of obstetric services, the authors recommend that hospitals and providers listen to the community-specific concerns of local residents. While the closure of rural obstetric departments often stems from issues outside of community control (e.g., low birth volume, staffing issues, financial reasons),^28^ rural residents acutely suffer the absence of obstetric services. In this community, the authors recommend that local entities (e.g., county social services, local nonprofits, faith-based organizations) help facilitate transportation services for pregnant women who need assistance with accessing obstetric care at urban hospitals. Since pregnant women in this community still frequented the local hospital when they were in labor or obstetric distress, local providers and the rural hospital should coordinate comprehensive communication efforts so that all residents know where pregnant women should go to receive proper care.

Moreover, these findings illustrate the importance of alternative and creative community health services for remote, rural communities that have experienced a loss of local obstetric services. While community-based health resources cannot fully ameliorate the loss of obstetric services in rural areas, investing in community-specific maternal health needs could promote positive outcomes for mothers and infants, even in the face of diminishing access to care.^5^ A quasi-experimental 30-year study in another region of rural Appalachia found that the strategic use of well-trained community health workers improved maternal access to prenatal care.^29^ Therefore, community health workers could potentially buffer the negative health effects of loss of obstetric services for women in rural Appalachian areas.^5^ Researchers should examine the impact of trained community health workers in rural Appalachian areas that no longer have easily accessible obstetric services. Additionally, researchers, healthcare advocates, and rural healthcare providers should call on policy makers to prioritize obstetric services in regions where women and infants are at increased risk for adverse birth outcomes. For example, the legalization of independent midwifery in NC could help ameliorate maternity care provider shortages in Appalachian western NC, as well as other rural regions of the state.^30^,^31^

Further, this analysis illustrates that oral history is an effective method to reveal various underlying, sociocultural factors that influence a medically underserved community’s perceptions of health care. The overarching research project and interview guide were not directly related to the topic of obstetric services, yet the flexible oral history method facilitated an opportunity for interviewees to discuss an issue that they perceived to be critically important to the health of their community. The historical nature of the interviews also allowed the authors to view the interviewees’ current healthcare needs within the context of how their community has changed over time. Through systematic analysis, the authors identified a salient community concern not drawn from a priori assumptions about what was important to health and well-being in this community. Thus, researchers, healthcare providers, and policymakers interested in understanding the broader implications of changes in healthcare services should consider how oral history can illuminate the events and issues that influence community members’ healthcare-seeking behaviors and attitudes.

Similar to most qualitative research, neither generalizability nor statistical representation was the goal of this study,^17^,^18^ yet a notable limitation of these findings is that they may not reflect the perspectives of local people of color. While there was evidence of theoretical saturation^18^ with this set of interviews, the fact that all participants were white could potentially obscure the healthcare experiences of people of color in this region of Appalachia. While people of color make up less than 8% of the population in the area where the interviews were conducted,^12^ research shows that structural racism operates in such a way that people of color in rural areas disproportionately bear the burden of insufficient healthcare resources and barriers to accessing care.^32^,^33^ Therefore, future health-related qualitative studies in rural Appalachia should purposively recruit people of color.

## IMPLICATIONS

As access to obstetric services in rural areas continues to decline,^3^,^5^ qualitative inquiry helps researchers and healthcare providers contextualize how the loss of obstetric services affects rural Appalachian residents’ attitudes and healthcare-seeking behaviors. In addition to perceived and actual increases in barriers to accessing care, the loss of services influenced this community’s mistrust of healthcare systems and nonlocal providers. Importantly, the results of this analysis highlight the need for community-specific interventions that mitigate the negative effects of declines in available services. These findings could aid in the development of interventions that effectively respond to all community members’ needs and concerns following the loss of obstetric services in rural Appalachian areas.

**SUMMARY BOX**

**What is already known on this topic?** The availability of inpatient obstetric services continues to decline in rural areas across the United States. In rural Appalachia, the termination of obstetric services threatens to exacerbate existing health disparities by increasing the risk of adverse birth outcomes for women and infants.

**What is added by this report?** Interviewees’ discontent with the loss of local obstetric services went beyond frustration with the financial burden of increased travel distance or trepidation about negative birth outcomes. The termination of services was associated with community members’ increased mistrust of healthcare systems, meaning that the termination of obstetric services could have health negative implications for many members of the community, regardless of their age, sex, or ability to bear children.

**What are the implications for future research?** These results offer formative research to support the development of interventions and policies that effectively respond to all community members’ needs following the loss of obstetric services in rural Appalachian communities. Future research should investigate the effectiveness of community health workers and community-specific strategies aimed at building trust with healthcare providers and improving rural access to care.

## Figures and Tables

**Table 1:** Data matrix example

Main code	Subcode	Summary of findings from subcode
Barriers to accessing care	Decline of available services	*Healthcare provider perspective:* Since the local hospital no longer offers obstetrical support, providers describe a curtailment of obstetric services provided by midwives and family medicine physicians in the area. They note an outflow of medical professionals who now have nowhere to practice.
		*Nonprovider perspective:* Nonproviders observe and lament the decline of obstetrical and general medical services in the area. They express astonishment and outrage that medical services available in the area decades ago can no longer be obtained at the local hospital. They argue the lack of available services poses a deep threat to the health of residents in their community.
